# Worry and rumination as a transdiagnostic target in young people: a co-produced systematic review and meta-analysis

**DOI:** 10.1080/16506073.2024.2369936

**Published:** 2024-06-26

**Authors:** Sarah J. Egan, Danyelle Greene, Thomas Callaghan, Shravan Raghav, Julia Funk, Theresa Badenbach, Samuel Talam, Georgia Kemp, Peter McEvoy, Thomas Ehring, Johannes Kopf-Beck

**Affiliations:** a enAble Institute, Faculty of Health Sciences, Curtin University, Perth, Australia; b Discipline of Psychology, Curtin School of Population Health, Faculty of Health Sciences, Curtin University, Perth, Australia; c Australian Institute for Business and Economics, University of Queensland, Brisbane, Australia; d Youth Programs, The Safe Place, Chennai, India; e Department of Psychology, LMU Munich, Munchen, Germany; f Amazing Minds Africa, Nairobi, Kenya; g Independent Lived Experience Expert Consultant, UK

**Keywords:** Worry, rumination, adolescents, anxiety, depression, psychosis, review

## Abstract

Repetitive Negative Thinking (RNT) encompasses both worry and rumination and is a transdiagnostic process. A systematic review and meta-analysis were co-produced with individuals with lived experience of anxiety and depression. The objective was to examine the efficacy of RNT-specific psychological interventions. Inclusion criteria were mean age or range 10–24 years, and including a measure of worry, rumination, RNT, anxiety, depression, psychosis, self-harm, or suicide. The Cochrane Risk of Bias measure was used. PsycInfo, Medline/PubMed, Scopus and Embase databases were searched on 24 April 2023. There were 16 studies with 20 RNT interventions, *N* = 1,183 participants, 79% female, mean pooled age = 20.08 years. There was a small effect of RNT interventions on anxiety symptoms (*g* = −0.43 to −0.47, 95% CI = −0.23 to −0.70). There were small-to-medium effects on depressive symptoms (*g* = −0.40 to −0.52, 95% CI = −0.19 to −0.84), and RNT (g = −0.59 to −0.71, 95% CI = −0.41 to −1.01). Most studies indicated some concerns for risk of bias. Transdiagnostic interventions that target RNT are efficacious in reducing symptoms of anxiety, depression, and RNT in young people. Further research is required to address younger age groups, psychosis, and different cultural contexts.

**Protocol Registration:**

PROSPERO (CRD42023408899).

Transdiagnostic approaches consider the role of processes maintaining multiple psychological disorders (Dalgleish et al., 2020). Worry and rumination are transdiagnostic processes (Ehring & Watkins, 2008; Zagaria et al., 2023), involved in anxiety, depression (Funk et al., 2022; McEvoy et al., 2018), psychosis (Hartley et al., 2014), suicidal ideation (Law & Tucker, 2018; Teismann et al., 2021) and self-harm (Dawkins et al., 2019). Worry involves thoughts about future problems, while rumination is past focused. Both worry and rumination are examples of “repetitive negative thinking” (RNT) (Stade & Ruscio, 2023). Definitions of RNT emphasise the tendency to get “stuck” in negative thinking (Ehring & Watkins, 2008). Given excessive RNT is a transdiagnostic process, it is an ideal target for early intervention for a range of psychological symptoms (Topper et al., 2010).

Interventions for RNT are based on addressing abstract styles of thinking and challenging metacognitions (e.g. Ehring & Behar, 2021; Ehring & Watkins, 2008; Wells, 2011). Metacognitions refer to positive and negative beliefs about thought processes and are theorised to maintain worry and rumination. For example, an individual may hold positive beliefs about worry as they think it can enable them to solve and avoid future problems, hence continue to worry as they think it is useful (Wells, 2011). Examples of RNT-specific interventions include rumination-focused Cognitive Behaviour Therapy (RFCBT; Watkins, 2016) and metacognitive therapy (MCT; Wells, 2011). MCT focuses on challenging the perceived benefits of RNT, and changing the process of thinking, including how intensely an individual engages with thoughts, rather than changing the content of thoughts. RFCBT also focuses on changing the process of thinking, through helping an individual learn to notice and change their thinking processes from an abstract style (e.g. “why am I such a failure?”) to a concrete style (e.g. “what exactly has led me to failing this time, what are the next steps I want to take?”). Interventions for RNT decrease psychological distress and improve quality of life with large effect sizes in adults (e.g. McEvoy et al., 2015).

The efficacy of RNT interventions in young people aged 14–24 years, with elevated symptoms of anxiety and depression, was examined by Bell et al. (2023). This review included interventions categorised as RNT focused, compared to non-RNT focused. There were 21 randomised controlled trials (RCTs) categorised as RNT focused interventions, which demonstrated a significant impact favouring intervention versus control at post-treatment, on anxiety (*g* = −0.42) and depression (*g* = −0.47). Additionally, Bell et al. (2023) concluded that interventions targeting RNT do not need to be long as higher treatment dose did not show better results. Bell et al.'s (2023) review was useful in highlighting the appeal of RNT interventions as brief, effective transdiagnostic interventions in young people. However, closer inspection of the interventions categorised as RNT focused raises questions over the specificity of the interventions included. Only around half of the interventions Bell et al. (2023) categorised as RNT focused appear to be RNT specific. The remaining studies classified as RNT focused were of standard or general psychological interventions not designed specifically to target RNT or based on a model of RNT as a maintaining mechanism of distress, such as eye movement desensitization (e.g. Lytle et al., 2002) and attention training to positive stimuli (e.g. Sass et al., 2017). Bell et al. (2023) did not outline any specific criteria that were used to judge whether an intervention was RNT-focused or non-RNT focused. Examples of interventions that the authors judged as non-RNT focused included CBT for insomnia, and self-monitoring. Further, Bell et al. (2023) did not report on insights from individuals with lived experience of anxiety and depression in their review.

Given previous reviews (Bell et al., 2023) did not report on insights from lived experience experts in their publication, it is important for a review to be co-produced with young people. It is imperative to integrate meaning from young people with lived experience of mental health problems through more extensive engagement consistent with co-production methods, including, for example, monthly meetings with lived experience experts over the course of a systematic review. Co-production with young people with lived experience of mental health problems is essential to produce a high-quality review of interventions for worry and rumination. Research in partnership with lived experience experts using co-production methods (Norton, 2021) is vital to inform best practice (Schleider, 2023). Co-produced research with individuals with lived experience of mental health problems can improve mental health service quality and delivery (Egan et al., 2023). Further, lived experience engagement is critical in improving intervention uptake (Schouten et al., 2022; Sunkel & Sartor, 2022).

The purpose of the current co-produced systematic review and meta-analysis was to examine the transdiagnostic efficacy of RNT interventions in young people aged 10–24 years. The four novel aspects of this review building on Bell et al. (2023) are as follows: (1) age range lowered to 10 years to examine evidence for early intervention, (2) outcomes extended beyond anxiety and depression to self-harm, suicide and psychosis, (3) inclusion of individuals with a “sub-threshold” diagnosis (i.e. elevated symptoms), in addition to young people with low to high symptom severity, which may also include prevention studies, and (4) co-production of the review with people with lived experience of mental health problems. Another purpose of the review was to understand through examination of moderators in which contexts, and for whom, RNT interventions appear to work.

The overall aim was to co-produce with young people a systematic review and meta-analysis to understand the efficacy of RNT interventions as a transdiagnostic early intervention. It was hypothesised that RNT interventions would result in significant decreases in symptoms of anxiety, depression, psychosis, and measures of suicide and self-harm.

## Method

### Co-production with young people with lived experience of mental health problems

Consistent with reviews highlighting the importance of young people co-producing reviews of interventions (e.g. Breen et al., 2023; Egan et al., 2022), collaboration included lived experience leads (SR, ST, GK) and a lived experience Youth Advisory Committee (YAC). The YAC comprised seven young people (19–29 years; gender 43% female, 29% male, 14% non-binary, 14% not reported) from Kenya (*n* = 2), India (*n* = 2), Australia (*n* = 2), and the United Kingdom (UK; *n* = 1). Lived experience leads identified young people for the committee, led YAC meetings, synthesised insights, and conducted key aspects of the research such as defining search terms, judging the degree of specific RNT content of interventions, and writing the publication. Individuals in the YAC signed consent forms prior to the first meeting, and the research was approved by the Curtin University Human Research Ethics Committee (HRE2023–0154). The YAC shared their insights into how worry and rumination were relevant to their lived experience of anxiety, depression, and psychosis. The YAC guided all stages of the research, for example, helping to create the search strategy and protocol, lay summary, infographic and video for dissemination.

### Search strategy and selection criteria

PsycINFO, Medline/PubMed, Scopus, Embase were searched on 24 April 2023, using the following search terms: depress* OR anxiety OR self-injury OR self-harm OR psychosis AND repetitive negative thinking OR negative thoughts OR worry OR rumination OR repetitive thinking OR perseverative cognition^1^ AND RCT OR randomised control trial. There were no date restrictions.

Articles were screened according to the following inclusion criteria: (a) peer-reviewed journal publication in English or German; (b) a controlled trial of worry/rumination/RNT intervention comparing to any form of control condition (including wait-list, no-treatment, placebo or active treatment comparison), (c) intervention(s) were focused specifically on worry, rumination or RNT (see Table 1 for definition), including both internet delivered and face-to-face interventions, (d) included a validated psychometric measure of worry, rumination or RNT and anxiety, depression, suicide, self-harm or psychosis, (e) participant mean age between 10 and 24 years (if mean age was not specified, an age range within these years), and (f) ethical approval and ascertainment of written informed consent in the published article. The exclusion criteria were as follows: (a) an intervention which is general, or another psychological approach not specifically stated as a treatment for worry/rumination/RNT (e.g. general cognitive behaviour therapy), and (b) open trials, qualitative studies, grey literature, dissertations, and unpublished studies.

**Table 1.** Definition of specific Repetitive Negative Thinking (RNT) interventions.

Inclusion: RNT specific intervention	Exclusion
Interventions specifically designed to target RNT, through the basis of either a theoretical or clinical model of RNT as a maintaining process of anxiety/depression/psychosis. Specific examples include: *Concreteness training* – specific form of Cognitive Behaviour Therapy (CBT) designed to target RNT. *Rumination Focused Cognitive Behaviour Therapy (RFCBT)* – specific form of CBT designed to target rumination. *RNT focused Acceptance and Commitment Therapy (ACT)* – specific form of ACT designed to target RNT. *Specific CBT techniques of worry journals* and *worry exposure* – techniques designed to reduce worry. *Worry disengagement training. RNT specific mental imagery training. Meta-Cognitive Therapy (MCT)* – Specific intervention designed to reduce RNT, included in this are component studies of MCT techniques including for example, *attentional control training* and *banning worry. Mindfulness Based Cognitive Therapy (MBCT)* – a specific intervention designed to target the way that individuals relate to repetitive and negative cognitions, theoretical models underpinning MBCT focus on the reduction of ruminative thinking.	Therapies which are not specifically designed for the purpose of targeting RNT, for example, where change in these processes may be a by-product rather than an intended target of the intervention, were excluded, for example, standard mindfulness and CBT interventions. Therapies where authors have not reported that the aim was to target worry, rumination or RNT, where for example a measure of RNT was used as an outcome but was not the main aim of the study. Therapies were general and not modified from their usual format for a range of other presenting problems, and therapies that were not specific to RNT or modified to address maintaining process of RNT through a theoretical model. Specific examples include: *General CBT* – this includes non-modified, typical CBT techniques of behavioural activation, cognitive restructuring, thought records. *General ACT* – this includes non-modified ACT, not modified to specifically have a focus on RNT. *General mindfulness* – standard mindfulness training not with the rationale to address worry or rumination, not MBCT. *General cognitive bias/attention bias modification* – where there is not a rationale to address worry/rumination or theoretical model where attention bias is a process in worry/rumination.

**Note**. These are examples of interventions to be included/excluded which were defined in the PROSPERO registration (CRD42023408899). Other interventions identified during the search which were RNT-specific of which we were not aware when outlining the registration, if judged as being RNT-specific, were included.

The reference list of Bell et al. (2023) was screened to identify any RNT-specific interventions that met our inclusion criteria which were not located by the database search. This resulted in 12 of the 21 articles included in the previous review being included in the current review according to our definition of an RNT-specific intervention (see Table 1 and supplementary materials).

### Procedure

The systematic review was registered with PROSPERO (CRD42023408899) on 28 March 2023. The primary rater (TC) screened 100% of titles/abstracts, with a random 30% screened independently by a secondary rater (TB), resulting in substantial agreement (Cohen’s *k* = 0.66; Landis & Koch, 1977). All full-text articles were screened by TC, with a random 30% screened independently by TB, which resulted in substantial agreement (Cohen’s *k* = 0.79; Landis & Koch, 1977). Consensus on final article inclusion was provided by SE and DG.

The YAC met in five online meetings. Young people co-produced the research including the proposal, search terms and registration, and dissemination outputs. In addition, young people were asked the following questions: *“do you see worry and rumination as relevant to your experience of, for example, anxiety and depression?”* and *“do you see an intervention for worry/rumination as relevant to you, would you be interested in doing it?”*. Further, young people discussed their interpretation of the findings from the systematic review and meta-analysis. They were invited to provide feedback via email when not able to attend meetings. YAC members received AUD$100 for each meeting.

### Risk of bias

The Cochrane Risk-of-Bias Tool V.2 (Sterne et al., 2019) was used due to following PRISMA guidelines (Page et al., 2021) which recommend this is assessed. The five domains were as follows: randomisation process, deviations from the intended interventions, missing outcome data, measurement of the outcome, and selection of the results. Categories are “low risk”; low risk for all domains; “some concerns”; some concerns in at least one domain; “high risk”; high risk in one domain or some concerns for multiple domains.

### Data analysis

Inter-rater reliability was calculated with Kappa coefficients in SPSS (Version 28; IBM Corp., 2021). The ranges are 0 = no agreement, .10–.20 = slight agreement, .21–.40 = fair agreement, .41–.60 = moderate agreement, .61–.80 = substantial agreement, .81–.99 = near perfect agreement, and 1 = perfect agreement (Landis & Koch, 1977).

Young people’s views expressed in the YAC meetings were summarised by SE using techniques of content analysis (Hsieh & Shannon, 2005), following a similar procedure to another co-designed review (Breen et al., 2023). Consensus was provided by SR and ST.

The data were analysed in a meta-analysis of between-groups effect sizes for the outcomes of worry, rumination, RNT, anxiety and depression. A Hunter-Schmidt Random Effects Model was used to pool effect sizes across studies for the primary outcomes of worry, rumination, RNT and psychological symptoms, due to significant variation across studies. To support the results of the random effects model we ran a Robust Bayesian meta-analysis (RoBMA) where we averaged across a set of 12 models to more accurately account for publication bias (see Maier et al., 2023). We assessed publication bias using Bayes factors and interpreted these based on the assumption that Bayes factors higher than one suggest evidence for publication bias. This method is superior to traditional methods such as Egger’s et al. (1997) test, which cannot distinguish between lack of publication bias and lack of evidence for publication bias (Bartoš et al., 2022). Several Robust Bayesian meta-analysis models account for publication bias, where the predictive utility of the two competing (i.e. effect vs null effect) hypotheses are judged using Bayes factors (BF10; van Doorn et al., 2021). Bayes factors should be considered on a continuum but for ease of understanding there are some general rules of thumb. First, a Bayes factor larger than one is considered supportive of the alternative hypothesis (i.e. an effect) and a Bayes factor less than one is supportive of the null hypothesis. Second, the level of evidence can be judged as follows: Bayes factors between 1 and 3 (or 1/3 to 1) are considered weak evidence, Bayes factors between 3 and 10 (or 1/10 to 1/3) are considered moderate evidence, and Bayes factors of 10 or larger (or smaller than 1/10) are considered strong evidence in favour (or against) a hypothesis (Jeffreys, 1961).

Heterogeneity was assessed via I^2^ statistics and Bayes factors. I^2^ estimates suggest the percentage of variance in effect sizes due to heterogeneity, and were categorized as low (0–40%), medium (41–60%), or high (61–100%; Moher et al., 2009). Bayes factors for heterogeneity were assessed, where Bayes factors larger than one indicate evidence in favour of heterogeneity. We anticipated heterogeneity to be substantial across studies for all outcomes due to different types of interventions, different duration, and format. Subgroups included: dose, experimental design versus multi-session intervention, outcome measure (i.e. worry, repetitive negative thinking, rumination), and type of intervention (therapist-led or self-help). The number of sessions and session duration was used to classify dose as short (<2 hours), medium (3–6 hours), or long (7+ hours) in total.

Effect sizes (Hedge’s g or dkorr) were calculated using pre- and post-test means from the experimental and control groups. We used the method described by Klauer (2001), where within-group effects are calculated for both the control and experimental groups and subtracted (i.e. intervention group effect-control group effect). Form three on the Psychometrica effect size calculators was used to compute dkorr (https://www.psychometrica.de/effect_size.html). A few studies only provided other statistics (e.g. f-values). For example, Pan et al. (2020) did not provide means and SD pre- and post-test for control and experimental groups but did provide between-group effect sizes that were controlled for baseline differences. These were converted to hedges g and imputed into the meta-analysis. Effect sizes were interpreted according to Cohen (1992), as small = 0.20–0.49, medium = 0.50–0.79, and large =>0.80, where a negative effect size indicates a decrease in psychological symptoms.

## Results

### Study characteristics

**Figure 1.**
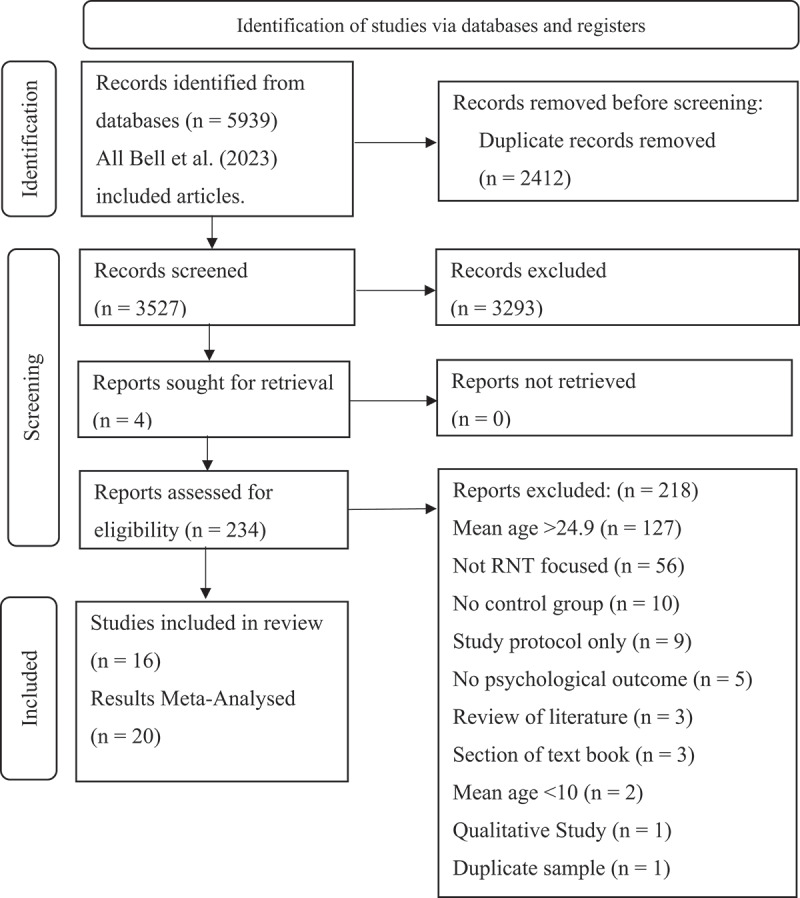
PRISMA diagram.

The search resulted in 3,527 studies after duplicate removal, of which 16 were included with 20 intervention groups (Figure 1). The studies were predominately conducted in the UK and Europe (38%), followed by Australia (19%), USA (19%), Colombia (12%), Iran (6%) and China (6%). All interventions were rated by GK as RNT-specific with consensus by SE (see supplementary materials). Most interventions (25%) were RFCBT (Watkins, 2016), followed by RNT-specific CBT (25%), MCT (19%; Wells, 2011), working memory training (WMT) (19%), and RNT-specific Acceptance and Commitment Therapy (ACT) (12%). RFCBT and MCT were described in the Introduction. RNT specific CBT includes interventions targeting RNT, for example, worry journals where an individual record outcomes of worry events. RNT-specific ACT uses techniques for “defusing” from stuck thinking. WMT involves computer tasks to improve retention and manipulation of information in working memory. Participants were mainly female (79%), and most (81%) were young adults (Tables 2 and 3).

*Selection flow chart.*

**Table 2.** Summary of characteristics across interventions.

	K^a^	N
*Country*		
Australia	3	153
China	2	106
Colombia	2	127
Germany	1	73
Iran	1	30
Netherlands	2	218
Romania	1	41
United Kingdom (UK)	5	307
United States of America (USA)	3	128
*Intervention Type*		
Rumination focused CBT (RFCBT)	6	474
Metacognitive therapy (MCT)	3	136
RNT specific CBT (RNT-CBT)	4	127
RNT specific ACT (RNT-ACT)	2	215
Working memory training (WMT)	5	231
Total number of participants	1183	

Note. ^**a**^number of intervention groups. Note. ACT = Acceptance and Commitment Therapy; CBT = Cognitive Behaviour Therapy; RNT = Repetitive Negative Thinking.

**Table 3.** Study characteristics.

First Author	N_inc_	Sample	Diagnosis	M Age	Female %	Length (total)	Country	Intervention	Individual or Group	I/E	Sessions	RNT Measure	Depression Measure	Anxiety Measure	ROB Rating
Bernal-Manrique (2020)	42	School students	–	14.52	71.4	3 hours, 45 min	Columbia	RNT focused ACT	Group	I	3 × 75 min	PTQ-C	DASS-21	BAI	some concerns
Cook (2019)	182	University students	–	20.41	83.4	6 hours	UK	RFCBT	Individual	I	6 × 60 min	RRS, PSWQ	PHQ-9	GAD-7	some concerns
Dereix-Calonge (2019)	85	University students	–	23.38	79	6 hours	Columbia	RNT focused ACT	Individual	I	3 × 120 min	PTQ	DASS-21	DASS-21	some concerns
Grol e(2018)	40	University students	–	23.17	86.67	2 hours	UK	WMT	Individual	2	N/A	PTQ, PSWQ	BDI	GAD-Q-IV	some concerns
Jacobs (2016)	33	rMDD adolescents	rMDD	15.55	57.58	8 hours	USA	RFCBT	Individual	I	8 × 45–60 min	RRS	RADS	–	some concerns
LaFreniere and Newman (2016)	51	University students	–	19.3	84.31	4 hours	USA	RNT specific CBT	Individual	I	Daily, 30 days	PSWQ,	–	GAD-Q-IV	some concerns
McDermott and Cougle (2021)	44	University students	–	19.02	92	3 hours	USA	RNT specific CBT: WDT	Individual	I	6 × 30 min	PSWQ	BDI-II	DASS-21	some concerns
McEvoy et al. (2014, 2017)	54	University students	–	23.61	80.2	12 min	Australia	MCT—Attention Training	Individual	E	1 × 12 min	–	–	STICSA	some concerns
Modini and Abbott (2017)	47	University students	SAD	19.89	74.5	90 min	Australia	RNT specific CBT	Individual	I	1 × 30 min	TQ	–	SAR	some concerns
Modini and Abbott (2018)	52	University students	SAD	19.94	67.9	20 min	Australia	MCT	Individual	I	1 × 20 min	TQ	–	SAR	some concerns
Mogoaşe et al. (2013)	41	University students	–	22.87	100	2 hours	Romania	RFCBT—Concreteness Training	Individual	I	7 × 15 min	RRS	BDI-II	–	some concerns
Pan et al. (2020)	106	University students	–	21.31	68.92	10 hours	China	WMT, E-WMT	Individual	E	21 × 40 min	RRS	–	STAI-T	high risk
Roberts et al. (2021)	85	University students	–	19.37	N/A	2 hours	UK	WMT	Individual	E	20 × 25 “trials”	RRS, PSWQ	PHQ-9		some concerns
Skodzik et al. (2018)	73	University students	–	22.02	84.82	2 hours	Germany	RNT specific CBT—Mental imagery	Individual	E	4 modules	PSWQ	–	BAI	some concerns
Topper and Emmelkamp et al. (2017)	218	Adolescent students	–	17.32	83.67	9 hours	Netherlands	RFCBT	Individual	I	6 × 90 min	RRS, PSWQ, PTQ	BDI-II	MASQ	some concerns
Zemestani et al. (2016)	30	University students	–	24.2	60.98	12 hours	Iran	MCT	Individual	I	8 × 40–60 min	CERQ	BDI-II	–	some concerns

**Note**: I = Randomised Controlled Trial (RCT) intervention study, E = experimental study, rMDD = major depressive disorder in remission, MDD = major depressive disorder, SAD = Social Anxiety Disorder, Length (total) = Total intervention time length, ROB = Risk of bias, RNT = repetitive negative thinking, ACT = acceptance and commitment therapy, RFCBT = rumination focused CBT, WDT = worry disengagement training, WMT = working memory training, E-WMT = electronic (internet delivered) working memory training, MCT = metacognitive therapy, PTQ-C = Perseverative Thinking Questionnaire—Children, RRS = Ruminative Response Scale, PSWQ = Penn-State Worry Questionnaire, PTQ = Perseverative Thinking Questionnaire, TQ = Thoughts Questionnaire, DASS-21 = Depression Anxiety Stress Scale − 21 items, RADS = Reynolds Adolescent Depression Scale, PHQ-9 = Patient Health Questionnaire − 9 items, BDI-II = Beck Depression Inventory − 2^nd^ edition, BAI = Beck Anxiety Inventory, GAD-7 = General Anxiety Disorder − 7 items, GAD-Q-V = Generalised Anxiety Disorder Questionnaire − 4, STICSA = State Trait Inventory for Cognitive and Somatic Anxiety, SAR = State Anxiety Rating, STAI-T = State Trait Anxiety Inventory—Trait Subscale, MASQ = Mood and Anxiety Symptom Questionnaire, CERQ = Cognitive Emotion Regulation Questionnaire.

### Risk of bias ratings

Most studies were categorised as having some concerns (93.75%), with the remainder as high risk (6.25%) (Table 3). There was moderate agreement (Cohen’s *k* = 0.55), between the primary (JF) and secondary rater (JKB) on risk of bias.

### Efficacy of repetitive negative thinking interventions

#### Effects on worry, rumination and repetitive negative thinking

The output of the RoBMA showed strong support for small-to-medium effects of the efficacy of RNT interventions on rumination (g_(hunter-schmidt)_ = −0.36; g_(RoBMA)_ = −0.32; Table 4) and worry (g_(hunter-schmidt)_ = −0.33; g_(RoBMA)_ = −0.30), and RNT (g_(hunter-schmidt)_ = −0.71; g_(RoBMA)_ = −0.59). The only noticeable difference between the random effects model and the RoBMA is on RNT, where the RoBMA gave a more conservative estimate. Random effects models suggested low to moderate-high heterogeneity (I^2^ = 20.86–64.08%), but RoBMA suggested we cannot be certain whether heterogeneity or publication bias is present (Table 4). There was strong evidence for the efficacy of RNT interventions on reducing global RNT, with a medium effect (g_(hunter-schmidt)_ = −0.51; g_(RoBMA)_ = −0.50). For global RNT, there was strong evidence for the presence of heterogeneity and moderate evidence for the absence of publication bias (Table 4).

**Table 4.** Pooled effects, heterogeneity, and publication bias results for all main analyses and sub-group analyses.

	k	Hunter-Schmidt g [95% CI]	RoBMA g [95% CRI]	BF_10_ effect	BF effect evidence	I^2^ Hunter-Schmidt	BF_10_ Heterogeneity	BF heterogeneity evidence	BF_10_ PB	BF PB evidence
**Repetitive negative thinking**										
**RNT**	**6**	-0.71 [−1.01, −0.41]***	**-0.59 [−0.99, 0.00]**	**24.57**	**Strong**_**h1**_	**64.08%**	**1.96**	**Weak**_**h1**_	**1.68**	**Weak**_**h1**_
**Worry**	**10**	-0.33 [−0.50, −0.16]***	**-0.30 [−0.48, 0.00]**	**16.68**	**Strong**_**h1**_	**33.88%**	**1.37**	**Weak**_**h1**_	**0.44**	**Weak**_**h0**_
**Rumination**	**8**	-0.36 [−0.53, −0.19]***	**-0.32 [−0.52, 0.00]**	**17.08**	**Strong**_**h1**_	**20.86%**	**0.90**	**Weak**_**h0**_	**0.51**	**Weak**_**h0**_
**Global RNT**	**19**	-0.51 [−0.73.–0.30]***	**-0.50 [−0.80, −0.21]**	**73.72**	**Strong**_**h1**_	**70.80%**	**44.93**	**Strong**_**h1**_	**0.16**	**Mod**_**h0**_
CBT (all)	10	−0.42 [−0.56, −0.28]***	−0.41 [−0.56, −0.26]	1370.04	Strong_h1_	0.00%	0.40	Weak_h0_	0.28	Mod_h0_
RFCBT	6	−0.41[−0.57, −0.25]***	−0.39 [−0.58, −0.17]	57.20	Strong_h1_	0.00%	0.57	Weak_h0_	0.34	Weak-mod_h0_
RNT-CBT	4	−0.44 [−0.71, −0.17]**	−0.34 [−0.70, 0.00]	5.33	Mod_h1_	0.00%	0.64	Weak_h0_	0.71	Weak_h0_
WMT	5	−0.21 [−0.50, 0.09]	−0.05 [−0.37, 0.00]	0.40	Weak_h0_	38.28%	1.25	Weak_h1_	1.18	Weak_h1_
Clinical	3	−0.41 [−0.75, −0.06]*	−0.22 [−0.71, 0.00]	1.43	Weak_h1_	0.00%	0.78	Weak_h0_	0.60	Weak_h0_
Non-clinical	16	−0.54 [−0.78, −0.30]***	−0.52 [−0.88, 0.00]	20.23	Strong_h1_	74.83%	229.10	Strong_h1_	0.19	Mod_h0_
Self-help	6	−0.37 [−0.54, −0.20]***	−0.35 [−0.55, 0.00]	34.87	Strong_h1_	0.00%	0.51	Weak_h0_	0.33	Mod_h0_
Therapist	5	−1.31 [−1.96, −0.65]***	−0.83 [−1.91, 0.00]	4.16	Mod_h1_	84.37%	5862.95	Strong_h1_	0.73	Weak_h0_
Experiment	8	−0.30 [−0.51, −0.08]**	−0.18 [−0.46, 0.00]	2.23	Weak_h1_	25.66%	0.96	Weak_h0_	1.41	Weak_h1_
Multi-session	11	−0.71 [−1.02, −0.40]***	−0.67 [−1.21, 0.00]	13.94	Strong_h1_	77.72%	317.52	Strong_h1_	0.19	Mod_h0_
Short (10 min-2hrs)	7	−0.20 [−0.41, 0.01]	−0.05 [−0.33, 0.00]	0.41	Weak_h0_	0.81%	0.72	Weak_h0_	1.03	Weak_h1_
Medium (3hrs-6hrs)	6	−0.61 [−0.98, −0.23]**	−0.46 [−1.05, 0.00]	4.81	Mod_h1_	72.73%	13.62	Strong_h1_	0.29	Mod_h0_
Long (7hrs+)	6	−0.81 [−1.21, −0.41]***	−0.63[−1.40, 0.00]	6.58	Mod_h1_	75.03%	4.59	Mod_h1_	0.63	Weak_h0_
**Depression**										
**Overall**	**11**	-0.52 [−0.84, −0.19]**	**-0.40 [−1.15, 0.00]**	**2.32**	**Weak**_**h1**_	**80.27%**	**150.04**	**Strong**_**h1**_	**0.16**	**Mod**_**h0**_
CBT (all)	7	−0.43 [−0.58, −0.27]***	−0.41 [−0.58, −0.22]	139.52	Strong_h1_	0.00%	0.51	Weak_h0_	0.37	Weak_h0_
RFCBT	6	−0.42 [−0.58, −0.26]***	−0.40 [−0.58, −0.16]	57.75	Strong_h1_	0.00%	0.58	Weak_h0_	0.38	Weak_h0_
Non-clinical	10	−0.52 [−0.86, −0.17]**	−0.37 [−1.22, 0.04]	1.73	Weak_h1_	82.02%	445.48	Strong_h1_	0.18	Mod_h0_
Self-help	5	−0.37 [−0.55, −0.19]***	−0.32 [−0.56, 0.00]	11.90	Strong_h1_	0.00%	0.64	Weak_h0_	0.56	Weak_h0_
Therapist	4	−1.34 [−2.13, −0.55]***	−0.49 [−2.05, 0.66]	1.58	Weak_h1_	86.45%	3064.93	Strong_h1_	0.57	Weak_h0_
Multi-session	9	−0.66 [−1.01, −0.31]***	−0.53 [−1.40, 0.00]	3.33	Mod_h1_	79.90%	22.18	Strong_h1_	0.23	Mod_h0_
Short (10 min-2hrs)	3	0.05 [−0.27, 0.37]	0.01 [−0.11, 0.18]	0.16	Mod_h0_	0.00%	0.52	Weak_h0_	0.62	Weak_h0_
Medium (3hrs-6hrs)	4	−0.42 [−0.66, −0.17]***	−0.33 [−0.65, 0.00]	6.23	Mod_h1_	27.36%	1.16	Weak_h1_	0.73	Weak_h0_
Long (7hrs+)	4	−1.21 [−1.93, −0.49]**	−0.48 [−2.00, 0.60]	1.48	Weak_h1_	86.86%	2991.07	Strong_h1_	0.57	Weak_h0_
**Anxiety**										
**Overall**	**13**	-0.47 [−0.70, −0.23]***	**-0.43 [−0.68, −0.16]**	**49.92**	**Strong**_**h1**_	**68.09%**	**2.15**	**Weak**_**h1**_	**0.22**	**Mod**_**h0**_
MCT	3	−1.34 [−2.50, −0.18]*	−0.42 [−1.85, 0.57]	1.44	Weak_h1_	88.11%	1798.11	Strong_h1_	0.59	Weak_h0_
CBT(all)	8	−0.40 [−0.56, −0.24]***	−0.38 [−0.55, −0.17]	62.47	Strong_h1_	13.62%	0.89	Weak_h0_	0.27	Mod_h0_
RFCBT	4	−0.44 [−0.67, −0.22]***	−0.39 [−0.67, 0.00]	9.42	Mod_h1_	41.30%	1.47	Weak_h1_	0.45	Weak_h0_
RNT-CBT	4	−0.29 [−0.56, −0.02]*	−0.12 [−0.50, 0.00]	0.84	Weak_h0_	0.00%	0.61	Weak_h0_	0.60	Weak_h0_
Non-clinical	11	−0.49 [−0.76, −0.23]***	−0.43 [−0.81, 0,00]	11.45	Strong_h1_	72.47%	4.82	Mod_h1_	0.23	mod_h0_
Self-help	5	−0.35 [−0.53, −0.17]***	−0.31 [−0.54, 0.00]	10.71	Strong_h1_	0.00%	0.71	Weak_h0_	0.37	Weak_h0_
Therapist	3	−1.28 [−2.10, −0.45]**	−0.49 [−1.95, 0.58]	1.64	Weak_h1_	86.21%	807.78	Strong_h1_	0.82	Weak_h0_
Experiment	5	−0.29 [−0.53, −0.05]*	−0.13 [−0.48, 0.00]	0.97	weak_h0_	0.00%	0.60	Weak_h0_	1.16	Weak_h1_
Multi-session	8	−0.59 [−0.92, −0.26]***	−0.46 [−1.14, 0.00]	4.03	Mod_h1_	77.58%	16.07	Strong_h1_	0.21	Mod_h0_
Short (10 min-2hrs)	5	−0.29 [−0.53, −0.05]*	−0.13 [−0.48, 0.00]	0.97	weak_h0_	0.00%	0.60	Weak_h0_	1.16	Weak_h1_
Medium (3hrs-6hrs)	5	−0.28 [−0.47, −0.09]**	−0.20 [−0.46, 0.00]	2.96	Weak_h1_	0.00%	0.47	Weak_h0_	0.40	Weak_h0_
Long (7hrs+)	3	−1.28 [−2.00, −0.56]***	−0.53 [−1.97, 0.52]	1.83	Weak_h1_	85.70%	294.42	Strong_h1_	1.43	Weak_h1_

**Note**. BF = Bayes factor, RoBMA = Robust Bayesian meta-analysis, RNT = repetitive negative thinking, CBT = Cognitive behaviour therapy, RFCBT = Rumination focused CBT, WMT = Working memory training, MCT = Metacognitive therapy, CI = confidence interval, CRI = Credibility interval. A Bayesian credibility interval is a 95% probability that the true effect would lie between this interval, given the current data. Experiment = Study with an experimental design, the intervention could be a single session or multiple session experiment. Multi-session = Study with intervention sessions that were conducted over 2 or more sessions but did not involve an experimental design. *p < .05, **p < .01, ***p < .001. Bayes factors between 1–3 (or 1/3–1) are considered weak evidence, Bayes factors between 3–10 (or 1/10 to 1/3) are considered moderate evidence, and Bayes factors of 10 or larger (or smaller than 1/10) are considered strong evidence in favour (or against) a hypothesis (Jeffreys, 1961)

Considering specific forms of RNT interventions, the evidence for a small effect (g_(hunter-schmidt)_ = −0.42 to −0.44; g_(RoBMA)_ = −0.34 to −0.41) of CBT interventions on global RNT was strong (RFCBT) to moderate (RNT-CBT; Table 4). Random effects models suggested low heterogeneity (I^2^ = 0.00%), but RoBMA suggested the evidence for the absence of heterogeneity with the current data is weak. There was weak to moderate evidence for the absence of publication bias across CBT intervention types. There was weak evidence in favour of a null effect of working memory training interventions on global RNT (g_(hunter-schmidt)_ = −0.21; g_(RoBMA)_ = −0.05), with little certainty as to whether heterogeneity or publication bias was present. There were not sufficient data points for MCT interventions to calculate a pooled effect, but two studies that tested MCT interventions and measured rumination support the efficacy of MCT on RNT (Modini & Abbott, 2018; Zemestani et al., 2016).

Self-help interventions had a small pooled effect on global RNT (g_(hunter-schmidt)_ = −0.37; g_(RoBMA)_ = −0.35), whereas therapist-led interventions produced a large effect (g_(hunter-schmidt)_ = −1.31; g_(RoBMA)_ = −0.83). However, there was strong evidence for the presence of heterogeneity among therapist-led interventions, indicating that particular therapist-led interventions might be more effective than others, but we cannot conclude which types.

**Figure 2.**
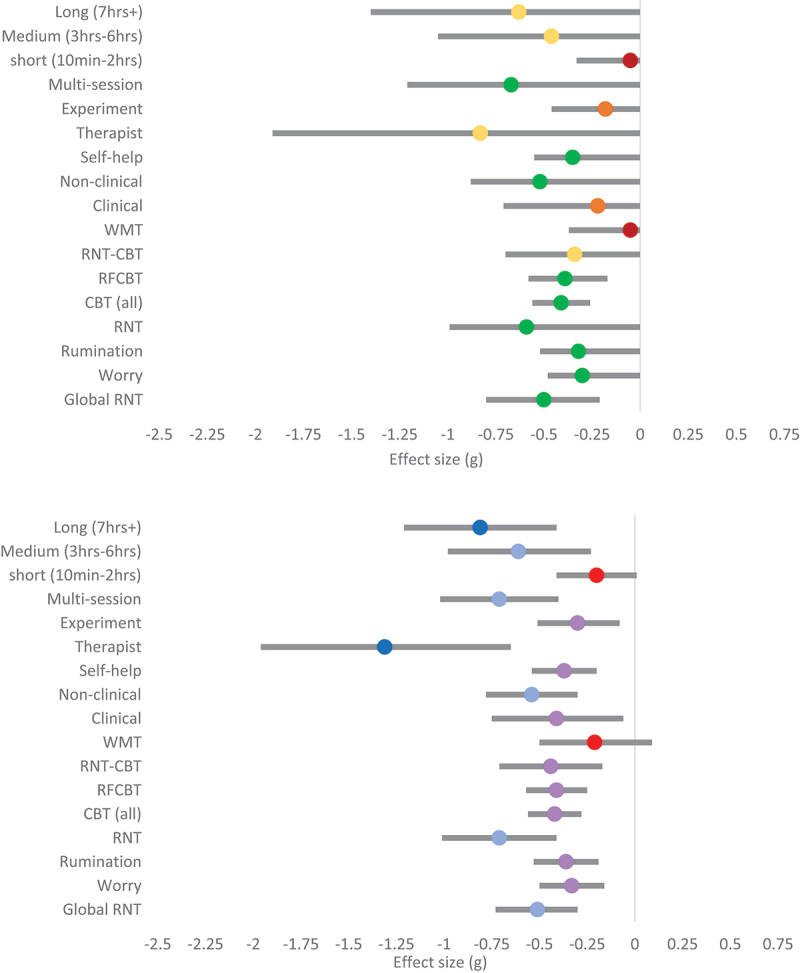
Forest plots for rumination.

There was weak evidence in favour of an effect of RNT experiments on global RNT, where the evidence for multi-session intervention on global RNT was strong (Table 4). Multi-session interventions produced a medium pooled effect on global RNT (g_(hunter-schmidt)_ = −0.71; g_(RoBMA)_ = −0.67), while experiments only produced a small effect (g_(hunter-schmidt)_ = −0.30; g_(RoBMA)_ = −0.18). Last, short interventions had little effect on reducing RNT (g_(hunter-schmidt)_ = −0.20; g_(RoBMA)_ = −0.05), whereas moderate and longer interventions had medium effects (g_(hunter-schmidt)_ = −0.61 to − 0.81; g_(RoBMA)_ = −0.46 to − 0.63; see Figure 2).

*The top panel shows subgroup pooled effects for repetitive negative thinking for the ROMBA model, with 95% credibility intervals. The dot represents the average (mean) pooled effect across models. Red =evidence in favour of H*_*0*_; *Orange = weak evidence in favour of H*_*1*_; *Yellow = moderate evidence in favour of H*_*1*_; *Green = strong evidence in favour of H*_*1*_. *The bottom panel shows subgroup pooled effects for repetitive negative thinking for the Hunter-Schmidt model, with 95% confidence intervals. Red = non-significant (p > .05); purple = small effect; light blue = medium effect; dark blue = large effect.*

#### Effects on anxiety

The output of the RoBMA showed strong support for the efficacy of RNT interventions on anxiety. The pooled effect was small (g_(hunter-schmidt)_ = −0.47; g_(RoBMA)_ = −0.43), with weak evidence for presence of heterogeneity and moderate evidence for the absence of publication bias. The random effects model demonstrated moderate–high levels of heterogeneity (I^2^ = 68.09%). Random effects models suggested MCT resulted in larger reductions in anxiety (g = −1.34), but RoBMA analysis suggested a more conservative, small effect (g = −0.42), with only weak evidence in favour of an effect. On closer inspection, one intervention (Zemestani et al., 2016) produced a very large effect that had a strong influence. Therefore, more studies with larger sample sizes are needed to confirm the efficacy of MCT on anxiety. The evidence for the efficacy of CBT interventions grouping together RFCBT and RNT specific CBT on anxiety was strong in favour of a small effect (g_(hunter-schmidt)_ = −0.40; g_(RoBMA)_ = −0.38). The evidence was less conclusive when considering RFCBT and RNT-CBT separately (Table 4).

Therapist-led RNT interventions appear to have a stronger effect on reducing anxiety (g_(hunter-schmidt)_ = −1.28; g_(RoBMA)_ = −0.49) compared to self-help interventions (g_(hunter-schmidt)_ = −0.35; g_(RoBMA)_ = −0.31). However, this comparison needs to be considered in light of RoBMA results suggesting that the current evidence is inconclusive for therapist-led interventions (BF_10_ = 1.64; Table 4). There were only three data points for therapist-led interventions, and there is strong evidence for large heterogeneity. Therefore, more therapist-led RNT intervention studies with larger sample sizes are needed to confirm the comparative efficacy between self-help and therapist-led interventions. Experimental design interventions were less effective at reducing anxiety (g_(hunter-schmidt)_ = −0.29; g_(RoBMA)_ = −0.13) compared to multi-session interventions (g_(hunter-schmidt)_ = −0.59; g_(RoBMA)_ = −0.46). However, the evidence in favour of an effect for multi-session intervention was barely moderate (BF_10_ = 4.03) and there was strong evidence for heterogeneity. This suggests that specific types of multi-session RNT interventions are more effective than others, but there were not enough studies to make any conclusions. Last, it appears that longer interventions (g_(hunter-schmidt)_ = −1.28; g_(RoBMA)_ = −0.53) are more effective than medium (g_(hunter-schmidt)_ = −0.28; g_(RoBMA)_ = −0.20) and short interventions (g_(hunter-schmidt)_ = −0.29; g_(RoBMA)_ = −0.13). However, RoBMA results indicated evidence was inconclusive, and there was strong evidence for heterogeneity among longer interventions.

#### Effects on depression

The output of the RoBMA showed weak support for the efficacy of RNT interventions on depression. The pooled effect was small-medium (g_(hunter-schmidt)_ = −0.52; g_(RoBMA)_ = −0.40) with strong evidence for presence of heterogeneity (I^2^ = 80.27%) and moderate evidence for the absence of publication bias. However, there was stronger evidence for an effect of CBT focused interventions on depression (g_(hunter-schmidt)_ = −0.43; g_(RoBMA)_ = −0.41). There was weak evidence in favour of the absence of publication bias and heterogeneity for CBT interventions (Table 4). There were not sufficient data points for MCT or WMT interventions to calculate a pooled effect of their efficacy.

**Figure 3.**
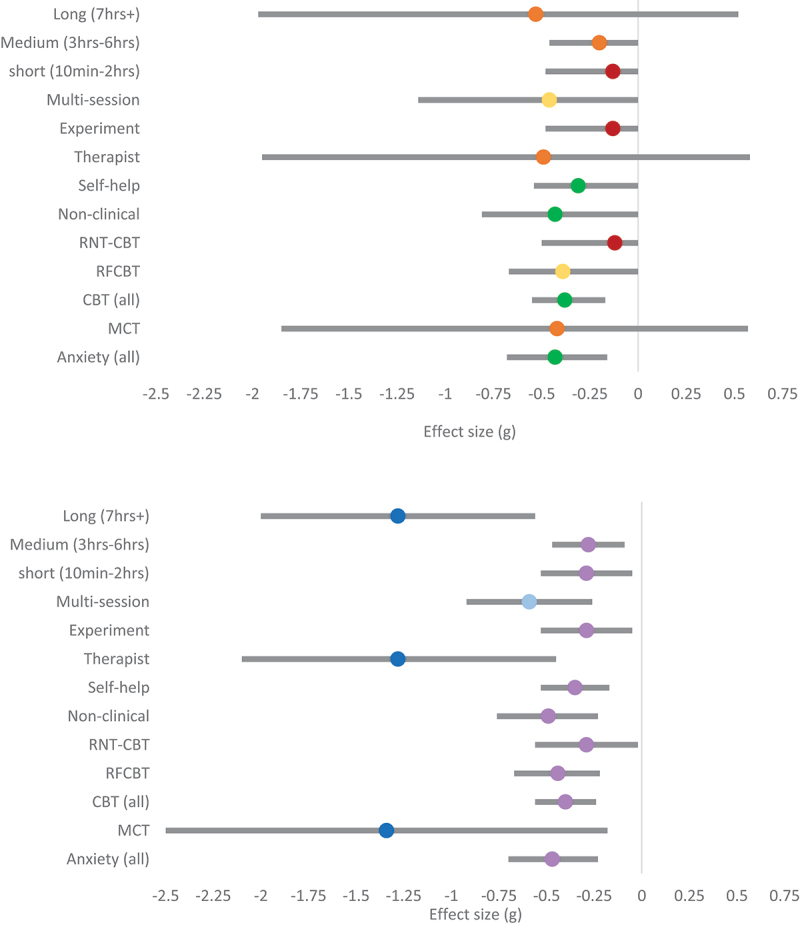
Forest plots for anxiety.

**Figure 4.**
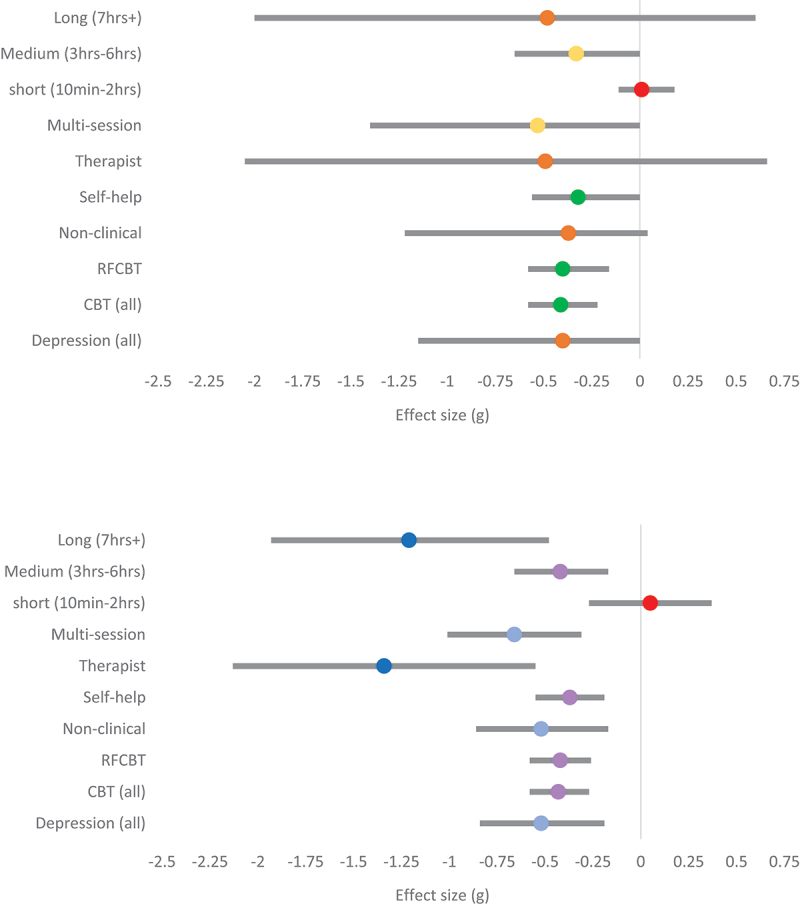
Forest plots for depression.

Therapist-led RNT interventions appear to have a stronger effect on depression (g_(hunter-schmidt)_ = −1.34; g_(RoBMA)_ = −0.49) compared to self-help interventions (g_(hunter-schmidt)_ = −0.37; g_(RoBMA)_ = −0.32). However, this comparison needs to be considered in light of RoBMA suggesting that the current evidence is inconclusive for therapist-led interventions (Table 4). There were only four data points for therapist-led interventions, and there is strong evidence for presence of heterogeneity among these effects. There were not enough data points to measure the effect of RNT experiments on depression. However, there was moderate evidence for an effect of multi-session RNT interventions in reducing depression. The pooled effect was medium (g_(hunter-schmidt)_ = −0.66 g_(RoBMA)_ = −0.53). There was strong evidence for the presence of heterogeneity and moderate evidence for the absence of publication bias. This suggests that specific types of multi-session RNT intervention might be more effective than others, but there were not enough studies to know which. Lastly, it appears that longer interventions (g_(hunter-schmidt)_ = −1.21; g_(RoBMA)_ = −0.48) are more effective than medium (g_(hunter-schmidt)_ = −0.42; g_(RoBMA)_ = −0.33) and short interventions (g_(hunter-schmidt)_ = 0.05; g_(RoBMA)_ = 0.01) in reducing depression. The evidence in favour of a null effect for short interventions is moderate, whereas the evidence in favour of an effect for medium and long interventions is moderate and weak, respectively. There is strong evidence for presence of heterogeneity in long interventions. See Figures 2–4 for a summary of all pooled effects.

*The top panel shows subgroup pooled effects for anxiety for the ROMBA model, with 95% credibility intervals. The dot represents the average (mean) pooled effect across models. Red = evidence in favour of H*_*0*_; *Orange = weak evidence in favour of H*_*1*_; *Yellow = moderate evidence in favour of H*_*1*_; *Green = strong evidence in favour of H*_*1*_. *The bottom panel shows subgroup pooled effect for anxiety for the Hunter-Schmidt model, with 95% confidence intervals. Red = non-significant (p > .05); purple = small effect; light blue = medium effect; dark blue = large effect.*

*The top panel shows subgroup pooled effects for depression for the ROMBA model, with 95% credibility intervals. The dot represents the average (mean) pooled effect across models. Red = evidence in favour of H*_*0*_; *Orange = weak evidence in favour of H*_*1*_; *Yellow = moderate evidence in favour of H*_*1*_; *Green = strong evidence in favour of H*_*1*_. *The bottom panel shows subgroup pooled effects for depression for the Hunter-Schmidt model, with 95% confidence intervals. Red = non-significant (p > .05); purple = small effect; light blue = medium effect; dark blue = large effect.*

### Young people’s views on worry and rumination

The YAC emphasised that worry and rumination were important to their lived experience of anxiety, depression, and psychosis. Young people commented about their experience saying, *“It’s our mind playing games with us, worrying about the future, you get anxious”* and *“I feel like worrying is a part of situations where I become anxious”*. Another young person said when they become aware they are worrying they think *“I don’t want to go down that route, down the rabbit hole of getting stuck in thoughts”*. Others said social media is an important part of their experience of worry and rumination, for example, *“Constant looking at social media becomes repetitive and obsessive”*. Some young people noted the difficulty in controlling repetitive thinking, for example, *“I have insight into rumination, yet I can’t control it, that makes it worse, you attribute that lack of control to yourself … that leads to negative self-esteem”*. Young people also talked about *“overthinking”*, e.g. *“I overthink a lot”*. Several people resonated with this, saying they engaged in *“overthinking a lot”.*

A discussion was also held about whether they would be interested in engaging in RNT interventions, and an example of MCT was discussed. All YAC members said they would be interested in treatments such as MCT for example, saying*“I can definitely see the benefits of this intervention for worry for my own experiences and for others I have known”*. One young person emphasised *“the earlier the better”*, and another said *“I also feel like learning such tools very early on in life could help us a lot. As kids, we pick up things much faster and it’s easier to form a habit at a young age than older”*. Another young person commented “*I think early interception of worry and rumination can in fact help avoid the outcomes of depression, anxiety, suicidal thoughts. There are so many instances where I find myself jumping from one bad thought to another in a fraction of a second.”* highlighting the importance of early intervention. Finally, young people said that treatments should be tailored according to cultural contexts saying, *“we are diverse, the treatments should be different across countries”.*

## Discussion

The aim of this co-produced review was to examine the efficacy of interventions for RNT. We also aimed to understand the views of young people about the relevance of RNT. Young people were unanimous in their views that worry and rumination were relevant to their lived experience of anxiety, depression, and psychosis. These insights are consistent with qualitative studies in young people (Oliver et al., 2015; Sloan et al., 2021) and adults (Joubert et al., 2022), where individuals described worry and rumination as relevant to their mental health problems. The young people who co-produced our review underscored that they were interested in engaging in RNT-specific interventions, that early intervention was key, and that they recommended intervention may differ across cultural contexts. We found evidence for the efficacy of RNT-specific interventions in reduction of worry, rumination, RNT, anxiety, and depression in young people aged 14–24 years. No studies were located including samples with a mean age between 10 and 14 years, or measures of psychosis, self-harm, or suicide.

The RNT-specific interventions in our review included interventions where the protocol was based on RNT specific material, leading to the exclusion of other interventions, largely cognitive bias modification studies, that were included in Bell et al. (2023). A strength of the current review was a stringent definition for what constituted an RNT-specific intervention, meaning conclusions can be drawn about interventions that are specifically designed to target RNT. Our findings of a small effect on anxiety (*g* = −0.43) were the same as Bell et al. (2023). We also found converging evidence for small-medium effects on depression (*g* = −0.40 to g = −0.52), similar to Bell et al. (2023) (*g* = −0.47). An important point to note when considering the small effects is that most studies included were in the context of prevention/early intervention, and most samples were non-clinical where smaller effect sizes are expected. However, examining prevention studies improves the generalisability of our results to young people in the community who are “sub-threshold” on psychological symptoms. Recent research has emphasised the promise of RNT interventions in prevention of mental health problems (Funk et al., 2023). Small effects in the context of prevention and early interventions have been argued to represent an important and effective outcome, where small effects can represent a large population benefit (O’Mara et al., 2023; Watson et al., 2016).

An important aspect of our review was an attempt to understand in which contexts, and for whom RNT interventions appear to work. We can conclude that short interventions of less than 2 hours duration are not recommended, as there was no impact on anxiety or depression. There was some evidence that interventions of a longer duration of 7 hours or more were more effective than medium length interventions. This result contrasts with Bell et al. (2023), who did not find higher treatment dose to be associated with larger effects. There was some evidence from subgroup analyses that therapist-led interventions demonstrated stronger effects than self-help, consistent with the literature where guided interventions have stronger effects than unguided self-help (Andersson, 2016). However, the evidence for therapist-led versus self-help interventions was largely classified as weak due to the small number of studies included in the analysis. Any conclusions regarding comparative efficacy between these intervention modes should be tentative until there are further studies available. Future meta-analyses should examine mode of intervention. Reviews should also seek to examine differences in age, for example, high school versus university students. We had an insufficient number of studies to perform this sub-group analysis, with only three studies including high-school students. Hence, our findings apply mainly to university students.

### Limitations and directions for future research

There were several limitations to our review. First, there were a relatively small number of studies, precluding examination of some moderators of change. We could not directly examine whether there were psychological mechanisms of change (e.g. change in metacognitive beliefs) that explained why RNT interventions resulted in reductions in anxiety and depression, as studies typically only reported symptom outcomes. However, given we observed medium reductions in RNT, the interventions did appear to target the proposed process. It is critical future treatment research examines causal reasons for how RNT interventions work, for example, which particular shared mechanisms, e.g. change in meta-cognitions, may be responsible for changes in RNT and symptoms across disorders. Consistent with previous reviews (Breen et al., 2023; Egan et al., 2022), we echo the need for examination of causal mechanisms underlying the efficacy of interventions. A further limitation was that no studies included measures of psychosis, self-harm, or suicide. Future research should broaden transdiagnostic outcomes beyond anxiety and depression, to include psychosis, self-harm, and suicide. It would also be useful to examine intervention at an earlier age, given most studies were in university students. A limitation to generalisability is most studies were conducted in high-income countries in the global North. More research is needed in low- and middle-income countries (LMICs) and different cultural contexts. This is especially relevant since young people from LMICs underscored the relevance of worry and rumination, but recommended treatments should be tailored across cultural contexts.

Future reviews could consider individual patient data meta-analysis to further understand the efficacy of RNT-specific interventions. Furthermore, many other interventions address transdiagnostic processes. Future research should investigate how efficacious RNT-specific interventions are compared to other transdiagnostic approaches. Future research should also examine which RNT-specific interventions are most efficacious for whom.

A final limitation is that while we decided to take a narrow approach to the definition of what constitutes an RNT intervention, with the rationale to provide a specific review of this mechanism, it is possible that in both our search terms and inclusion/exclusion criteria that some studies which may inform understanding of RNT interventions were missed. For example, one study we did not include and is relevant that showed promising results was a pilot feasibility trial based on the Laval model of Dugas et al. (1998) of intolerance of uncertainty in adults applied to a child and adolescent sample (Perrin et al., 2019). Furthermore, there are a range of general CBT programs for anxiety (e.g. Cool Kids; Lyneham et al., 2003), which have been demonstrated in a review by Hudson et al. (2015), which combined data from RCTs and uncontrolled trials, to result in a 58% remission rate for GAD in children and adolescents at follow-up. While we decided to take a narrow approach to examine what we considered to be RNT specific interventions to inform the effects of mechanism-specific interventions, future reviews could consider a broader approach and include all CBT interventions, where there is a substantial cognitive component, to determine the efficacy of a wider range of interventions than we included and compare to our findings. Partly, the findings of our review and Bell et al. (2023) were similar, although a novel aspect of our review, besides a more selective and distinct definition of RNT-specific treatments, was reporting on the views of young people with lived experience of mental health challenges who guided our review. Our rationale for a selective definition of RNT interventions was that a distinct definition may help to inform understanding of specific mechanisms of change. However, it is an important question for future reviews to consider whether examining a wider range of interventions is a more informative approach than a specific focus of RNT interventions.

## Conclusion

RNT-specific interventions are efficacious in reducing anxiety and depression in young people. Clinicians should consider delivery of RNT interventions for longer than 2 hours. Further research is required to examine the comparative efficacy between self-help and therapist-led interventions. Further research is also required with broader psychological outcomes and across cultural contexts.

## Supplementary Material

Supplemental Material

## Note

1. The inclusion of the search term “perseverative cognition” is an example of how our search strategy was coproduced with lived experience experts.
