# Supplementary material for: Worry and rumination as a transdiagnostic target in young people: a co-produced systematic review and meta-analysis
Source: Cogn Behav Ther. 2024 Jun 26;54(1):17–40. doi: 10.1080/16506073.2024.2369936 (PMC11627211; doi:10.1080/16506073.2024.2369936)
Supplement: Supplemental Material [file SBEH_A_2369936_SM0403.docx]

**Supplementary Materials.**

**Information on classification of RNT specific interventions compared to Bell et al. (2023).**

An RNT specific intervention was determined through coding of interventions classified by Bell et al. (2023) as RNT focused by SE with consensus provided by GK, JKB and JF. The 21 articles included by Bell et al. (2023), were read at full text level coded as yes/no RNT focused intervention(s). This resulted in 12 of the 21 articles (57%)^[[1]](#footnote-1)^ classified as RNT focused by Bell et al. (2023) also being judged an RNT specific intervention. The study by McIntosh and Crino (2013) included in Bell et al. (2023) was judged as an RNT specific intervention, however, was not included in the current meta-analysis as it did not meet our criteria of including a control group. The remaining 9 articles (43%)^[[2]](#footnote-2)^ classified as RNT focused by Bell et al. (2023) were judged as not being RNT specific interventions, according to our definition of an RNT specific intervention (see Table 1 of manuscript).

**Supplementary Materials**

Table 1. Intervention descriptions and degree of repetitive negative thinking (RNT) content for each intervention study.

| **Study** | **Intervention condition** | **RNT related procedure content** | **Non RNT-related content and control** | **RNT related content estimated >50%** |
| --- | --- | --- | --- | --- |
| Bernal-Manrique et al. (2020) | RNT focussed Acceptance and Commitment Therapy (ACT). | ACT protocol (adapted). Three, 75-minute sessions were RNT related content. | None stated for intervention. Control condition was waitlist. | Yes |
| Cook et al. (2019) | Web-based Rumination-focused Cognitive Behavioural Therapy (RFCBT). | Guided RFCBT compared with unguided RFCBT and a waitlist (usual care) control. Intervention translated and adapted from Topper et al. (2017). Six, 1-hour modules, each split into 3 or 4 sessions with 1 to 2 weeks recommended per module for practice of the techniques. | None stated for intervention.  Waitlist control. | Yes |
| Dereix-Calonge et al. (2019) | Acceptance and Commitment Training (ACT) focused on disrupting RNT. | Six, 1-hour sessions of RNT-focused ACT intervention. ACT intervention was designed to be web-based; the sessions of this study were conducted face to face. | None stated for intervention. Waitlist control. | Yes |
| Grol et al. (2018) | Working Memory Training (WMT) for worry symptoms. | Adaptive WMT combined with sham Cognitive Bias Modification (CBM) compared to CBM combined with sham WMT, or sham WMT combined with sham CBM. | None stated. | Yes |
| Jacobs et al. (2016) | Rumination-Focused Cognitive Behaviour Therapy (RFCBT). | RFCBT participants received weekly, 45–60-minute intervention facilitated by therapist (first author) for eight weeks. | Not stated. Assessment only control group. | Yes |
| LaFreniere & Newman (2016) | CBT Worry Journal. | Participants recorded worry content, predictions about the future, completed ratings for each worry, marked whether the worry came true, and reported if the outcome was better than, as bad as, or worse than expected after the worry event passed. | None stated. Control was non-worry related thought logging. | Yes |
| McEvoy et al. (2017) | Attention Training Technique (ATT) from Metacognitive Therapy (MCT). | A 12-minute audio recording of how to shift attention. | Compared to Progressive Muscle Relaxation and thought wandering control. | Yes |
| McDermott & Cougle (2021) | Worry Disengagement Training (WDT). | Six 30-minute computerized sessions. Each session consisted of four worry and four disengagement writing periods. | Waitlist - Completed the same baseline session but without the WDT intervention. | Yes |
| Modini & Abbott (2017) | Brief cognitive restructuring & exposure during task. | Testing was conducted over three sessions, each one-week apart. First testing session: all participants informed they would be required to complete a three-minute videotaped speech task. Participants were guided in challenging negative thoughts. | Not stated - cognitive restructuring intervention focused on rumination but paper does not report this in detail. | Yes |
| Modini & Abbott (2018) | Metacognitive therapy technique 'detached mindfulness'. | Intervention based on work by Professor Adrian Wells, involved providing a definition of negative rumination, eliciting the advantages and disadvantages of negative pre-event rumination, with any perceived advantages challenged. | Not stated. | Yes |
| Mogoaşe et al. (2013) | Concreteness training. | Seven scheduled daily sessions designed to last about 15 minutes. Used a total of five positive and five negative written scenarios. Every scenario was presented to participants on a standard form. | Not stated. | Yes |
| Pan et al. (2020) | Mobile applications-based Working Memory Training (WMT) & Emotional working memory training (EWMT). | WMT and EWMT completed daily 30 training blocks for 40 min for 21 days. WMT required to remember the colour and position of the block on a 3x3 matrix. Participants stated whether the colour and position of the current block was the same as the forward ‘nth’ character. “N” represented working memory load and performance. EWMT materials were emotionally negative faces. | Placebo control included baseline measures (questionnaires, cognitive tasks, and resting EEG) | Yes |
| Roberts et al. (2021) | Cognitive control training (CCT): Computerised adaptive working memory updating training (WMT). | Computerised adaptive WMT in reducing RNT (task became increasingly difficult as performance improved). Intervention 20 training sessions over 28 days of either (a) WMT using neutral stimuli, (b) WMT using negative stimuli, or (c) non-adaptive WMT. | Non-adaptive working memory updating training (control). | Yes |
| Skodzik et al. (2018) | Training in Mental Imagery (TMI) | Compared the effect of a novel training in mental imagery on various worry-related outcomes to a control training in verbal thinking and a waiting-list control group. | Wait list control & non-therapeutic training in verbal thinking control. | Yes |
| Topper et al. (2017) | RNT focussed CBT | Participants showing elevated RNT were randomly allocated to a 6-week cognitive-behavioural training delivered in a group, via the internet, or to a waitlist control condition. | Not stated. | Yes |
| Zemestani et al. (2016) | Meta-Cognitive Therapy (MCT) | The treatment groups received 8 weekly MCT sessions based on Well's manual. Each session and homework trained participants to use an auditory attention training technique. MCT included how to identify and challenge negative and positive metacognitive beliefs about rumination and worry and the uncontrollability of thoughts. | Each MCT session manual content not explicitly stated. No non-related RNT content described. Compared to Behavioural Activation. | Yes |

1. Bernal Manrique et al. (2020); Grol et al. (2018); La-Freniere & Newman (2016); McDermott et al. (2021); McEvoy et al. (2017); McIntosh & Crino (2013); Modini & Abbott (2017, 2018); Mogoase et al. (2013), Skodzik et al. (2018), Topper et al. (2017); Zemestani et al. (2016). [↑](#footnote-ref-1)
2. de Voogd et al., (2017a,b); Kocovski et al. (2019); Lytle et al. (2002); McIndoo et al. (2016); Sass et al. (2017); Teng et al. (2019); Vrijsen et al. (2019); Yang et al. (2016). [↑](#footnote-ref-2)
